# CMTM7 knockdown increases tumorigenicity of human non-small cell lung cancer cells and EGFR-AKT signaling by reducing Rab5 activation

**DOI:** 10.18632/oncotarget.5732

**Published:** 2015-10-26

**Authors:** Baocai Liu, Yu Su, Ting Li, Wanqiong Yuan, Xiaoning Mo, Henan Li, Qihua He, Dalong Ma, Wenling Han

**Affiliations:** ^1^ Center for Human Disease Genomics, Department of Immunology, Key Laboratory of Medical Immunology, Ministry of Health, School of Basic Medical Sciences, Peking University, Beijing, China; ^2^ Department of Clinical Laboratory, Beijing Jishuitan Hospital, Beijing, China; ^3^ Department of Clinical Laboratory, Peking University People's Hospital, Beijing, China; ^4^ Peking University Medical and Health Analysis Center, Beijing, China

**Keywords:** NSCLC, CMTM7, EGFR, AKT, Rab5

## Abstract

The dysregulation of epidermal growth factor receptor (EGFR) signaling has been well documented to contribute to the progression of non-small cell lung cancer (NSCLC), the leading cause of cancer death in the world. EGF-stimulated EGFR activation induces receptor internalization and degradation, which plays an important role in EGFR signaling. This process is frequently deregulated in cancer cells, leading to enhanced EGFR levels and signaling. Our previous study on CMTM7 is only limited to a brief description of the relationship of overexpressed CMTM7 with EGFR-AKT signaling. The biological functions of endogenous CMTM7 and its molecular mechanism remained unclear. In this study, we show that the stable knockdown of CMTM7 augments the malignant potential of NSCLC cells and enhances EGFR-AKT signaling by decreasing EGFR internalization and degradation. Mechanistically, CMTM7 knockdown reduces the activation of Rab5, a protein known to be required for early endosome fusion. In NSCLC, the loss of CMTM7 would therefore serve to sustain aberrant EGFR-mediated oncogenic signaling. Together, our findings highlight the role of CMTM7 in the regulation of EGFR signaling in tumor cells, revealing CMTM7 as a novel molecule related to Rab5 activation.

## INTRODUCTION

CKLF-like MARVEL transmembrane domain-containing family (CMTM) is a family of proteins that link classical chemokines and the transmembrane-4 superfamily [1, 2]. In humans, CMTMs are encoded by nine genes: *CKLF* and *CMTM1-8*. CMTM proteins play important roles in the immune system and male reproduction as well as tumorigenesis [3–8]. *CMTM7* is a 3p22.3 tumor suppressor that is down-regulated or absent in esophageal tumor tissues with promoter methylation and loss of heterozygosity [8]. CMTM7 restoration in esophageal squamous cell carcinoma (ESCC) cell lines inhibits cell growth, promotes epidermal growth factor receptor (EGFR) internalization, and suppresses the AKT signaling pathway [8]. An immunohistochemistry assay with tissue microarray indicated that CMTM7 is also down-regulated in lung cancer [8]. Moreover, Sarit Aviel-Ronen et al. reported that CMTM7 is down-regulated in lung cancer tissues compared with normal tissues [9]. Liu et al. found that aberrant CMTM7 expression is a unique prognostic factor for NSCLC survival [10]. These data indicate that CMTM7 may play a crucial role as a tumor suppressor in lung cancer development.

Lung cancer is the leading cause of cancer death worldwide, and approximately 85% of lung cancers are non-small cell lung cancer (NSCLC) [11, 12]. EGFR overexpression or constitutive activation occurs in approximately 60% of NSCLC cases and is correlated with poor prognosis [13]. One important mechanism of EGFR regulation is the internalization of activated EGFR [14]. EGFR endocytosis is a multistep process, including receptor internalization at the plasma membrane, sorting in early endosomes, transport to late endosomes, uptake in multi-vesicular bodies and degradation in the lysosomes [15]. The process of EGFR internalization and degradation is generally known as receptor down-regulation and is considered an important cellular strategy for signal attenuation [16, 17]. The GTPase Rab5 plays a critical role in EGFR internalization, vesicle trafficking and fusion with early endosomes [18, 19]. Deletion of Rab5 inhibits the transport of EGFR and consequently causes sustained EGFR signaling and delayed EGFR degradation [20]. Similar to other G proteins, Rab5 cycles between an inactive GDP-bound state and an active GTP-bound form. When Rab5 is activated, it recruits cytosolic factors, such as EEA1 and Rabaptin-5, to promote endosome docking and fusion [21]. Aberrant Rab5 activation leads to alterations in endosome fusion, EGFR signaling and degradation [22, 23]. Thus, the activation of Rab5 must be coordinated for the maintenance of proper trafficking.

The role of CMTM7 in tumorigenic signaling and development is currently unclear. Our previous study showed that CMTM7 overexpression reduces EGFR-AKT signaling in esophageal carcinoma cells, but the molecular details in this progress are not yet clear. Importantly, EGFR is a key target for NSCLC therapy. Thus, we investigated the relevance of CMTM7 loss in NSCLC with *in vitro* and *in vivo* models. In this study, we provide novel insights into the contributions of CMTM7 to regulating EGFR signaling. We used lentiviral expression constructs to knock down endogenous CMTM7 in NSCLC cells. The stable knockdown of CMTM7 promoted AKT signaling, leading to enhanced tumor growth and metastasis. Further, CMTM7 knockdown delayed EGFR internalization and degradation. Consistent with these results, CMTM7 knockdown significantly enhanced the epidermal growth factor (EGF)-induced EGFR-AKT signaling cascade and cell migration. Importantly, we report for the first time that CMTM7 knockdown reduces Rab5 activation. Thus, the loss of CMTM7 in NSCLC serves to sustain aberrant EGFR-mediated oncogenic signaling.

## RESULTS

### CMTM7 knockdown promotes NSCLC cell growth

**Figure 1:**
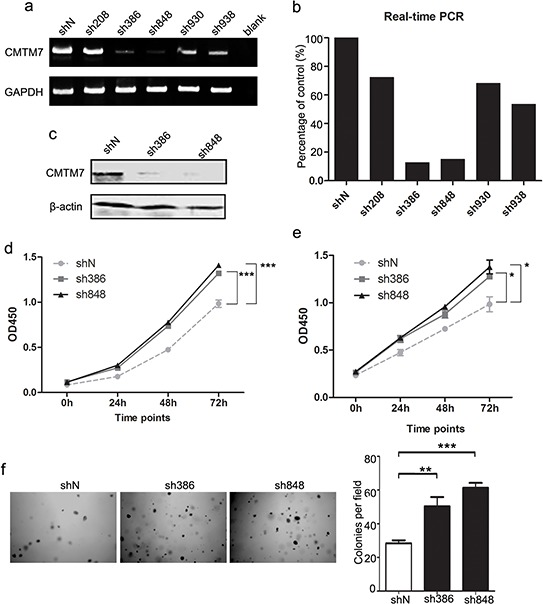
CMTM7 knockdown promotes A549 and HCC827 cell proliferation **a, b.** A549 cells were transfected with five distinct lentiviral CMTM7 shRNAs, named according to the last three numbers of the cloning item—sh208, 386, 848, 930 and 938—or the matching control non-targeting shRNA (shN). CMTM7 mRNA expression was analyzed using semi-quantitative (a) and real-time RT-PCR (b). **c.** A549 cells were transfected with CMTM7 shRNA (sh386, sh848) or the matching control non-targeting shRNA (shN). CMTM7 protein expression was analyzed by western blotting. **d, e.** CCK8 assay of control and CMTM7-knockdown NSCLC cells, A549 (d) and HCC827 cells (e). Cell numbers were determined every 24 h. Data are expressed as the mean ± s.d (*P < 0.05, ***P < 0.001). **f.** Representative images of cell colonies in control and CMTM7-knockdown A549 cells (magnification, × 100). Colonies with > 100 cells were quantified. Data are presented as the mean ± s.d (**P < 0.01, ***P < 0.001).

To examine the biological functions of endogenous CMTM7 in NSCLC, we generated A549 cells stably expressing lentiviral short hairpin RNA (shRNA) to knock down CMTM7. Five different nucleotide sequences were designed for shRNA. The two sequences with the best knockdown efficiency were selected for the subsequent experiments and named according to the last three numbers of the cloning item: sh386 and sh848 (typically more than 80% knockdown) (Figures 1a–1c). The effect of CMTM7 knockdown on cell growth was determined according to a CCK8 assay. Both sh386 and sh848 cells exhibited significantly higher proliferation rates (1.35-fold and 1.44-fold at 72 h, respectively) compared to control cells (Figure 1d). To further validate this result, we tested the effects of CMTM7 knockdown on the growth of HCC827 cells, a common NSCLC cell line. Both sh386 and sh848 lentivirus exhibited good knockdown efficiency in HCC827 cells (data not shown). In agreement with the A549 cell results, CMTM7-knockdown cells exhibited an average 1.36-fold increase in cell growth compared with control cells at 72 h (Figure 1e). To determine whether CMTM7 influenced anchorage-independent growth, we performed soft-agar colony formation assays on CMTM7-knockdown and control A549 cells. As shown in Figure 1f, CMTM7 knockdown significantly increased the soft agar colony formation ability of A549 cells (~ 2-fold) (Figure 1f). These data indicate that CMTM7 knockdown promotes tumor cell growth in both anchorage-dependent and independent conditions, underlying the crucial role of CMTM7 in NSCLC cell growth.

### CMTM7 knockdown enhances NSCLC cell migration

**Figure 2:**
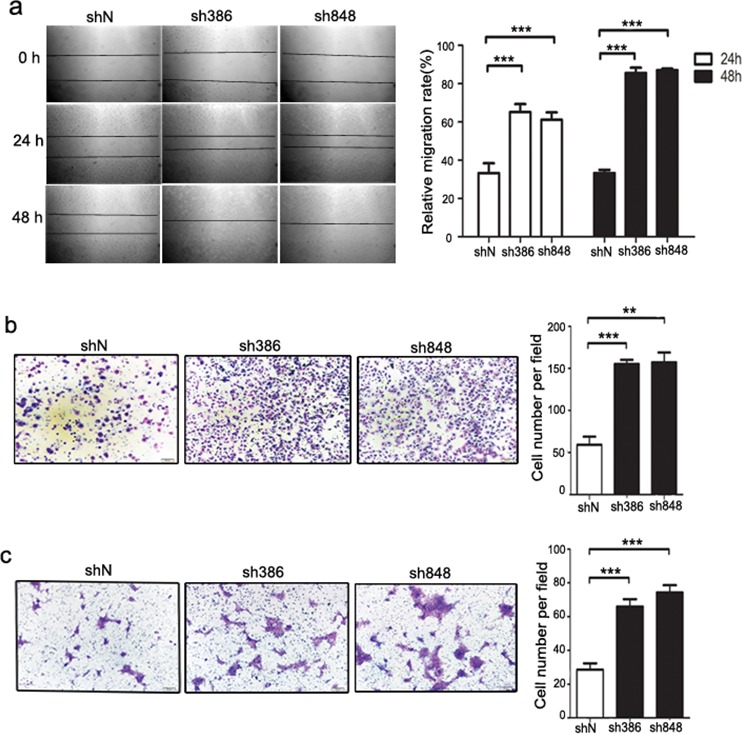
CMTM7 knockdown enhances the migration of A549 and HCC827 cells **a.** Wound-healing assay of control and CMTM7-knockdown A549 cells. Confluent monolayers of control or CMTM7-knockdown cells were wounded and incubated for the indicated times. The relative migration rate at each time point (t) was calculated of changed mean gap distance (MGD) relative to the initial MGD (T0) with the formula [MGD(T0)- MGD(t)/MGD(T0)]x100%. Data are representative of three independent experiments (magnification, × 100) and are presented as the mean ± s.d (***P < 0.001). **b, c.** Transwell migration assay of control and CMTM7-knockdown NSCLC cells, A549 (b) and HCC827 cells (c). Photos were taken after 24 h of incubation, and the cells were stained with crystal violet (magnification, × 100). The graph indicates the mean ± s.d and *P* values of the number of cells per five random high-power fields (magnification, × 400) counted from three independent experiments (**P < 0.01, ***P < 0.001).

To further determine whether CMTM7 is associated with the progression of NSCLC, we evaluated its effect on cancer cell migration ability via a wound-healing assay. As shown in Figure 2a, CMTM7 knockdown significantly enhanced A549 cell migration. CMTM7-knockdown cells exhibited full wound closure after 48 h, whereas for the control cells, a distance between wound edges remained visible. To confirm the involvement of CMTM7 in cell migration, we measured cell migration according to a Transwell assay. The number of migrated CMTM7-knockdown A549 cells was significantly higher than that of control cells (~ 2.64-fold) (Figure 2b). Similarly, CMTM7 knockdown promoted the HCC827 cell migration according to a Transwell assay (~ 2.45-fold) (Figure 2c). These results suggest that CMTM7 knockdown enhances NSCLC cell migration.

### CMTM7 knockdown promotes NSCLC cell metastasis *in vivo*

**Figure 3:**
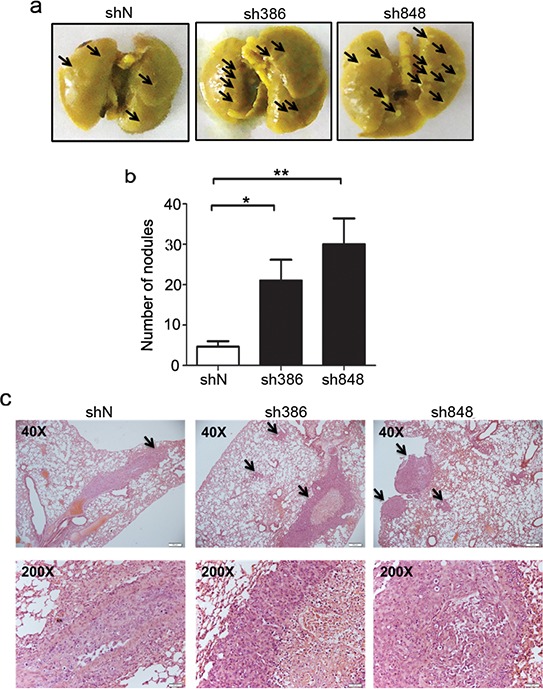
CMTM7 knockdown enhances lung metastasis of A549 cells **a.** Representative images of mouse lungs. Arrows indicate pulmonary metastatic tumor nodules. **b.** Quantitative results (6 mice per group) of pulmonary metastatic tumor nodules at 6 weeks after injection. Data are expressed as the mean ± s.d (**P < 0.01). **c.** Representative hematoxylin and eosin-stained lung sections containing metastatic foci. Pictures were taken under a microscope at × 40. Arrows indicate pulmonary metastatic tumor.

The above *in vitro* studies indicated that CMTM7 knockdown might enhance the *in vivo* metastasis of NSCLC cells. Therefore, we analyzed the metastatic tumor nodules formed in the lungs of NOD-SCID mice after tail vein inoculation with CMTM7-knockdown or control A549 cells. As expected, the tumor formation in the lungs of mice injected with CMTM7-knockdown cells was more apparent than that of mice injected with control cells (Figure 3a). Moreover, the number of metastatic nodules in the lungs was significantly higher in the mice injected with CMTM7-knockdown cells (Figures 3a–3c). These results suggest that endogenous CMTM7 is a negative regulator of NSCLC metastasis.

### Effects of CMTM7 knockdown on growth and migration via PI3K/AKT-dependent signaling

**Figure 4:**
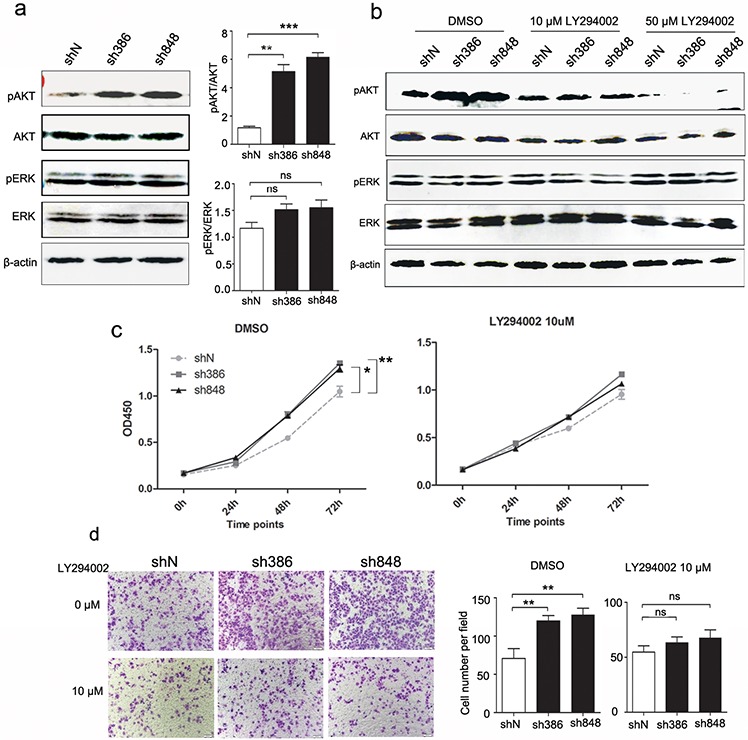
Effects of CMTM7 knockdown on growth and migration via PI3K/AKT-dependent signaling **a.** CMTM7 knockdown enhances AKT phosphorylation. Protein lysates of control and CMTM7-knockdown cells were immunoblotted with antibodies as indicated. The normalized densitometry data using ImageJ software are the means of three independent experiments and are presented as the mean ± s.d (**P < 0.01, ***P < 0.001; ns, not significant). **b.** The inhibiting efficiency of LY294002 on PI3K/Akt signaling was tested by western blotting. **c.** Control and CMTM7-knockdown A549 cells were treated with LY294004 (10 μM) or DMSO as a vehicle control, and cell proliferation was analyzed via CCK8 assay. Data are representative of three independent experiments. Data are expressed as the mean ± s.d (*P < 0.05, **P < 0.01). **d.** Control and CMTM7-knockdown A549 cells were treated with LY294004 (10 μM) or DMSO, and cell migration was analyzed via Transwell migration assay. Photos were taken after 24 h of incubation, and the cells were stained with crystal violet (magnification, × 100). The graph indicates the mean ± s.d and *P* values of the number of cells per five random high-power fields (magnification, × 400) counted from three independent experiments (**P < 0.01; ns, not significant).

The PI3K/AKT and Ras/ERK signaling pathways play crucial roles in the initiation and development of many cancers, including NSCLC. Our previous studies indicated that CMTM7 overexpression inhibits the phosphorylation of AKT but not ERK in ESCC cells. We next investigated the effects of CMTM7 knockdown on AKT and ERK signaling in NSCLC cells. As shown in Figure 4a, under normal growth conditions, CMTM7 knockdown in A549 cells substantially enhanced AKT phosphorylation (~ 4.84-fold) but had no obvious effects on pERK levels, which is in accordance with our previous report.

To assess whether the promoting effects of CMTM7 knockdown on cell growth and migration depend on PI3K/AKT pathway, we treated A549 cells with the PI3K inhibitor LY294002 and conducted CCK8 and Transwell assays. As shown in Figure 4b, LY294002 treatment blocked the PI3K/AKT pathway in a dose-dependent manner, but not ERK. Notably, LY294002 at 10 uM reversed the enhanced AKT phosphorylation following CMTM7 silencing. In addition, LY294002 (10 μM) suppressed the malignant behavior in CMTM7-knockdown cells to the levels similar as control cells treated with this inhibitor (Figures 4c–4d). These results suggest that the effects of CMTM7 knockdown on NSCLC cell aggressiveness *in vitro* are mediated by PI3K/AKT dependent signaling pathways.

### CMTM7 knockdown results in enhanced EGF-induced migration and signaling

**Figure 5:**
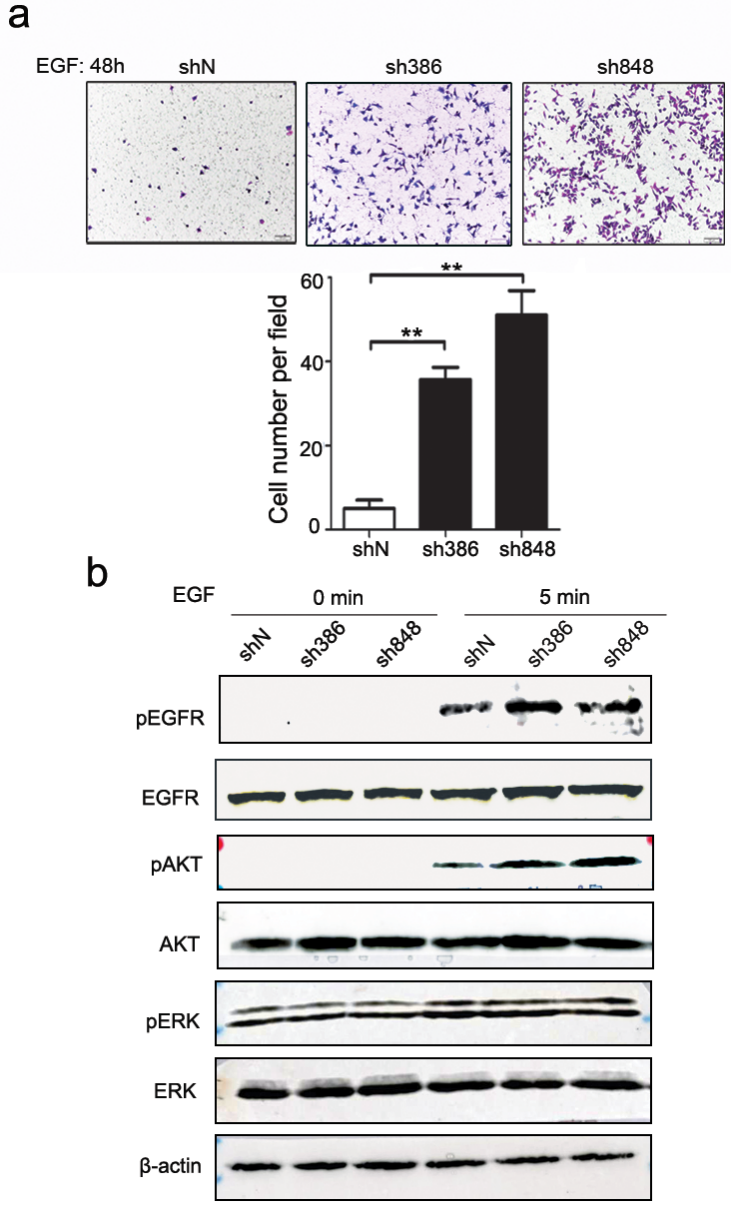
CMTM7 knockdown results in enhanced EGF-induced migration and signaling **a.** Effects of CMTM7 knockdown on EGF-induced migration via Transwell assay. Photos were taken after 48 h, and cells were stained with crystal violet (magnification, × 100). The graph indicates the mean ± s.d and *P* values of the number of cells per five random high-power fields (magnification, × 400) counted from three independent experiments (**P < 0.01, ***P < 0.001). **b.** The effect of CMTM7 knockdown on EGFR-dependent signaling was determined using western blotting of EGF-exposed cells for phospho-EGFR, phospho-AKT, and phospho-ERK. β-actin was used as an internal control.

The above results reveal that CMTM7 knockdown promotes serum-induced NSCLC cell migration. Serum is a relatively undefined mixture that mediates complex changes in signaling. Our previous study showed that CMTM7 restoration in ESCC cells reduces EGFR signaling. Because EGF is the most studied growth factor in NSCLC, we further investigated the effects of CMTM7 knockdown on EGF-mediated migration. As shown in Figure 5a, CMTM7 knockdown significantly enhanced EGF-induced cell migration (~ 8.67-fold). We next assessed the effects of CMTM7 knockdown on EGF-stimulated EGFR activation, as determined by the major autophosphorylation site Y1173, and phosphorylation of the downstream signaling targets AKT and ERK. As shown in Figure 5b, CMTM7 knockdown increased the phosphorylation of EGFR and AKT but not ERK. These findings suggest that CMTM7 regulates cell migration through the EGFR-AKT signaling pathway.

### CMTM7 knockdown attenuates EGFR internalization and delays its degradation

**Figure 6:**
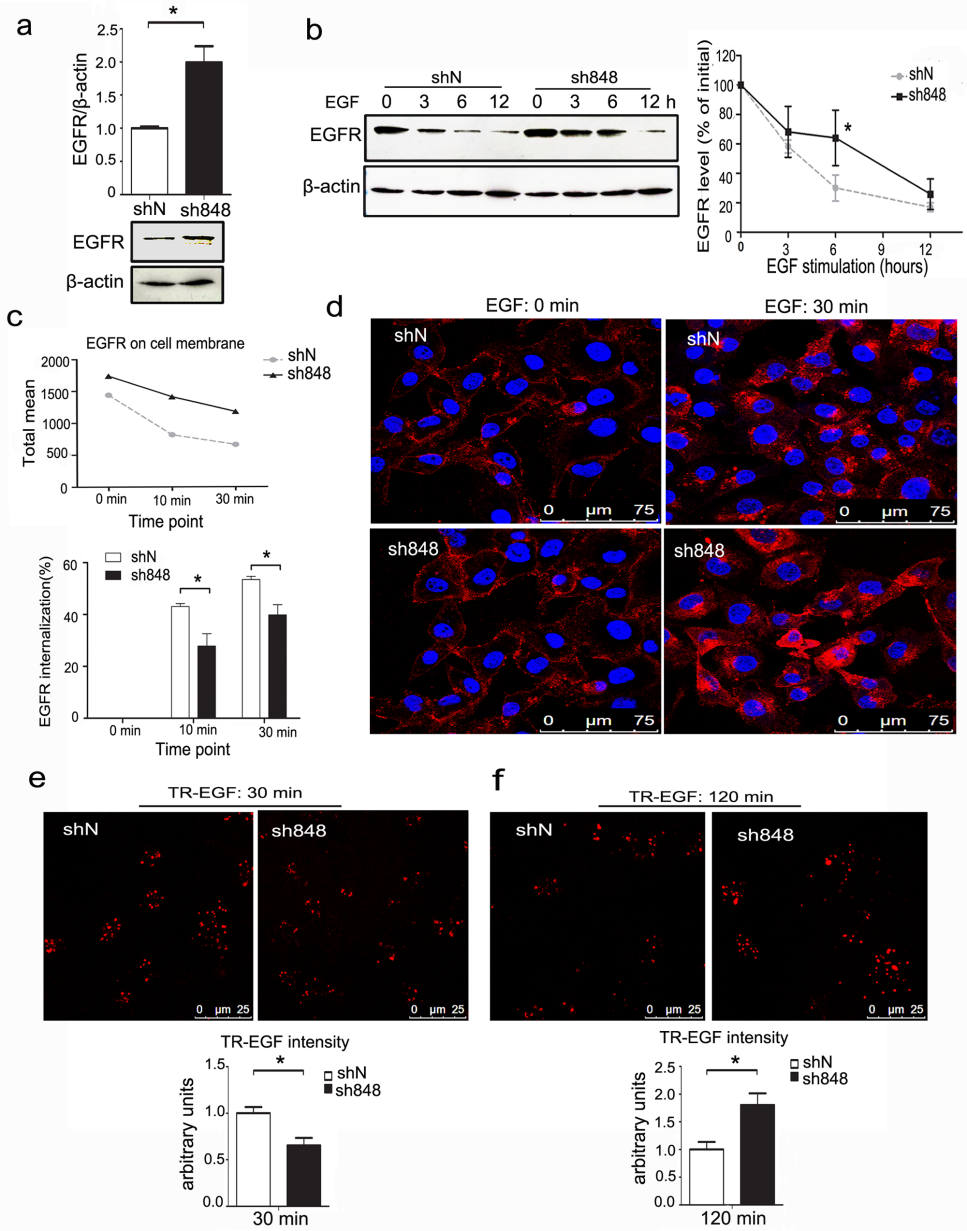
CMTM7 knockdown attenuates EGFR internalization and delays its degradation **a.** Control and CMTM7-knockdown A549 cells were cultured in complete medium, and western blots of the cell lysates were probed with antibodies against the indicated proteins. The normalized densitometry data using ImageJ software are the means of three independent experiments and are presented as the mean ± s.d (*P < 0.05). **b.** Control and CMTM7-knockdown A549 cells were pretreated with cycloheximide (CHX) (100 μg/ml) for 1 h prior to treatment with EGF (100 ng/ml) in the presence of CHX for the indicated times, and the cell lysates were subjected to immunoblotting with the indicated antibodies. The normalized densitometry data using ImageJ software are means of three independent experiments and are presented as the mean ± s.d (*P < 0.05). **c.** FACS analysis of EGFR surface levels following time-dependent EGF stimulation of CMTM7-knockdown A549 cells. EGFR internalization was quantified via mean fluorescence intensity. The graph shows the percentage of internalization at indicated time points. **d.** EGFR immunofluorescence analysis of CMTM7-knockdown A549 cells following EGF stimulation. Control and CMTM7-knockdown A549 cells were plated on coverslips, starved for 16 h, stimulated with 100 ng/ml EGF for 30 min, fixed, blocked and incubated with a rabbit anti-EGFR polyclonal antibody and TRITC-conjugated secondary antibody. The cells were washed prior to analysis via confocal microscopy. **e, f.** Control and CMTM7-knockdown A549 cells were treated with 1000 ng/ml Texas Red-EGF for 30 min at 4°C, followed by additional incubation for 30 (e) or 120 min (f) at 37°C. In each case, the fluorescence intensity of Texas Red-EGF was measured in 50 cells. Texas Red-EGF fluorescence intensity was quantified using LEICA QWin software and is presented as the mean ± s.d (*P < 0.05).

Because CMTM7 knockdown by sh848 is more effective at enhancing EGFR and AKT phosphorylation, sh848 cells were used for all subsequent experiments. To elucidate the molecular mechanism mediating CMTM7 function in EGFR signaling, we determined the effect of CMTM7 knockdown on EGFR protein levels. In normal culture, CMTM7 knockdown increased total EGFR levels (Figure 6a). Because ligand-induced degradation is the primary mechanism that controls EGFR levels, we next explored whether CMTM7 affects EGFR degradation in response to EGF. To directly assess degradation of EGFR, we used cycloheximide (CHX) to block protein translation. As shown in Figure 6b, the initial rate of EGF-induced EGFR degradation was significantly slower in the CMTM7-knockdown compared with the control cells. However, the receptors eventually underwent degradation to an extent similar to the control cells.

Because EGFR internalization is linked to its degradation, we next performed flow cytometry and measured the internalization of EGFR by quantifying the EGFR remaining on the cell surface after EGF stimulation. As shown in Figure 6c, the mean fluorescence intensity of EGFR on the cell surface in CMTM7-knockdown A549 cells incubated with EGF for the indicated times was higher than that of control cells. In addition, the EGFR internalization rates were reduced in CMTM7-knockdown cells relative to control cells. Further immunofluorescence-based analysis showed that CMTM7-knockdown cells had more EGFR on the cell surface after EGF stimulation for 30 min (Figure 6d). These results suggest that CMTM7 knockdown delays EGFR internalization.

To visualize the effects of CMTM7 knockdown on EGFR internalization and degradation, we investigated Texas Red-EGF (TR-EGF) internalization using confocal microscopy. After 30 min of TR-EGF stimulation, the amount of TR-EGF internalization was lower in CMTM7-knockdown cells (Figure 6e). Finally, after 120 min of TR-EGF stimulation, we observed that CMTM7-knockdown cells had much higher TR-EGF signaling levels than control cells (Figure 6f). Collectively, these results further verified that CMTM7 knockdown not only delays internalization but also suppresses EGFR degradation.

### CMTM7 localizes to Rab5 and EEA1-positive microdomains on the limiting membrane of early endosomes

**Figure 7:**
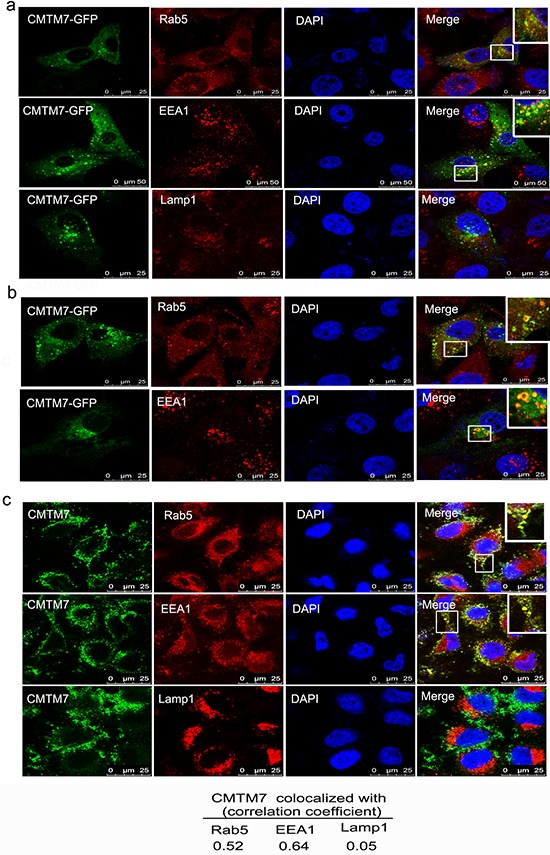
CMTM7 localizes to Rab5 and EEA1-positive microdomains on the limiting membrane of early endosomes **a.** A549 cells were transfected with CMTM7-GFP for 24 h prior to fixation in 3% PFA and immunostained with antibodies against Rab5, EEA1 or Lamp1. Insets show magnification of colocalization of CMTM7-GFP with early endosomes. **b.** Insets show that CMTM7-GFP localizes to Rab5 and EEA1-positive “doughnut-shaped” compartments characteristic of unusually large endosomes. **c.** Endogenous CMTM7 localizes to early endosome and enlarged early endosome. Insets show magnification of CMTM7 induced enlarged endosomes. All endocytic markers are shown in red. Nuclei are visualized by DAPI (blue). The yellow color indicates colocalization. Bar, 25 μm. The Pearson's and Manders' overlap coefficients were derived with LEICA QWin software and are the average of ten individual cells.

To investigate the mechanism of CMTM7 on EGFR trafficking, colocalization studies were initially performed with the early endosome protein DsRed-Rab5 and late endosome protein Lamp1-mCherry in A549 cells. Overexpressed CMTM7-GFP clearly colocalized with DsRed-Rab5-positive structures without colocalization with Lamp1-mCherry (Supplementary Figure S1). Interestingly, CMTM7-GFP overexpression caused a striking redistribution of early endosomes to form a larger concentration of puncta (Supplementary Figure S1). We then used a panel of antibodies that recognize early and late endosomes, finding CMTM7-GFP was predominantly associated with Rab5 and EEA1 without colocalization with Lamp1 (Figure 7a). Although CMTM7-positive early endosomes were not obviously enlarged, CMTM7-GFP occasionally localized to Rab5 and EEA1-positive “doughnut-shaped” compartments characteristic of unusually large endosomes (Figure 7b). As early endosome fusion can cause the appearance of unusually large endocytic structures, it is indicated that CMTM7 may promote early endosome fusion/enlargement. To further characterize the localization of CMTM7 in the endocytic pathway, we used one monoclonal antibody against endogenous CMTM7 which can be used in immunofluorescence. Figure 7c showed that the staining pattern of endogenous CMTM7 is similar to that of CMTM7-GFP. Importantly, endogenous CMTM7 can obviously colocalize with early endosomes and enlarged early endosomes, but not late endosomes, suggesting a role of CMTM7 in early endosomes.

### CMTM7 knockdown delays early endosome fusion by reducing Rab5 activation

**Figure 8:**
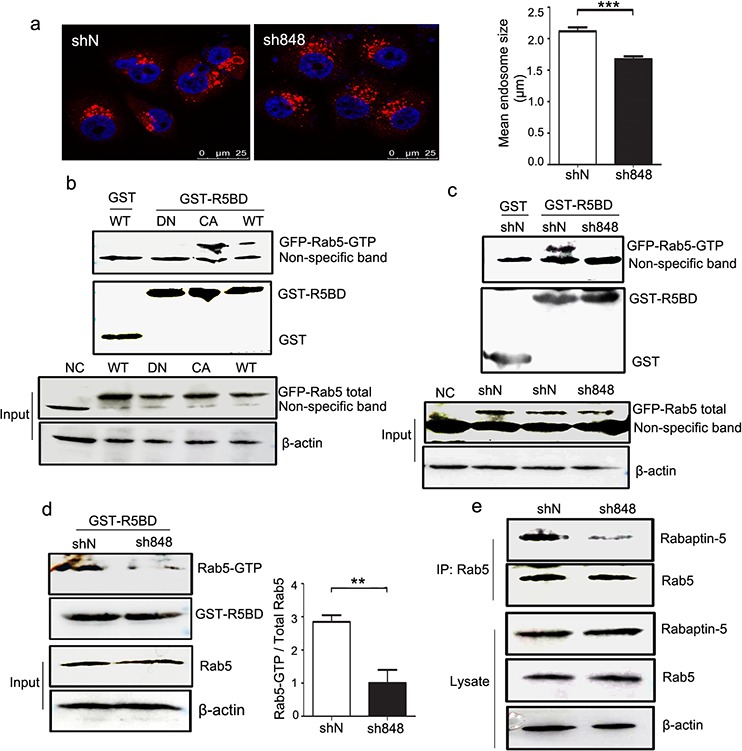
CMTM7 knockdown inhibits endosome fusion by reducing Rab5 activation **a.** Control and CMTM7-knockdown A549 cells were fixed in 3% PFA and stained with an anti-EEA1 (red) antibody. The different sizes of EEA1-positive endosomes were captured by confocal microscopy. Nuclei are shown in blue. Bar, 25 μm. LEICA QWin software was used to automatically measure the size of each EEA1-positive spot (***P < 0.001). **b.** HEK293T cells were transfected with GFP-tagged Rab5-WT, Rab5-DN, Rab5-CA, or nothing (negative control, NC). At 36 h post-transfection, the cell lysates were subjected to pull-down with GST or GST-R5BD beads. Western blots of precipitates and total cell lysates were probed with GFP antibody. **c.** Control and CMTM7-knockdown A549 cells were transfected with GFP-tagged Rab5-WT. After 36 h, the cells were subjected to a similar procedure and analysis. **d.** The lysates of control and CMTM7-knockdown A549 cells were subjected to GST or GST-R5BD pull-down, and endogenous Rab5-GTP levels were analyzed via western blotting. Relative Rab5-GTP levels are shown on the right. Data are presented as the means of three independent experiments (**P < 0.01). **e.** Control and CMTM7-knockdown A549 cells were lysed, and Rab5 was immunoprecipitated. The immunoprecipitated proteins were resolved on SDS-PAGE, and the levels of Rab5 and Rabaptin5 were detected by western blotting. Data are representative of three independent experiments.

We next analyzed endosome formation in both CMTM7-knockdown and control cells. In CMTM7-knockdown cells, EEA1 staining was more disperse and was present on the smaller structures rather than on the larger clustered structures typical of control cells (Figure 8a). The association of EEA1 with endosomes and subsequent endosome fusion events was regulated by Rab5 activity [24]. Rabaptin5, a direct Rab5 effector, specifically interacts with Rab5 in its active GTP-bound state [25]. To determine whether CMTM7 knockdown inhibits Rab5 activation, we performed pull-down assays using the GST-tagged Rab5-binding domain of Rabaptin5 (residues 739–862, R5BD) responsible for Rab5-GTP binding [25]. The specificity of GST-R5BD binding to active Rab5 was shown by its more efficient binding to Rab5-CA compared with Rab5-WT and lack of binding to a dominant-negative Rab5 S43N mutant (Rab5-DN) (Figure 8b). Importantly, GST-R5BD pulled down less active exogenous or endogenous Rab5 from CMTM7-knockdown cells than from control cells (Figures 8c–8d). Consistent with the GST-R5BD pull-down assays, the coimmunoprecipitation interaction of Rab5 with Rabaptin5 was weaker in CMTM7-knockdown cells (Figure 8e). These results suggest that CMTM7 knockdown can delay endosome fusion by reducing Rab5 activation.

## DISCUSSION

Our previous study on CMTM7 was limited to a brief description of its relation to EGFR-AKT signaling [8]. How CMTM7 affects EGFR-AKT signaling remained unclear. In this study, we demonstrated that the stable knockdown of CMTM7 in NSCLC cells delays EGFR degradation by attenuating its internalization in response to EGF stimulation, which in turn promotes EGFR-AKT signaling and EGF-induced cell migration. Moreover, CMTM7 is localized to Rab5/EEA1-containing early endosomes as well as to enlarged early endosomes. Importantly, CMTM7 knockdown delays early endosome fusion by reducing Rab5 activation. Notably, CMTM7 knockdown also results in a decrease in EGFR ubiquitination (Supplementary Figure S2). Although EGFR ubiquitination is not necessary for its internalization, it is required for EGFR lysosomal sorting [26, 27], suggesting that CMTM7 knockdown delays EGFR degradation at least in part by suppressing receptor ubiquitylation. Overall, these findings identify CMTM7 as a new regulator of Rab5 activation in the intracellular trafficking network that controls EGFR signaling.

Overexpression, mutations, deletions and the production of autocrine ligands contribute to aberrant EGFR activation in NSCLC [28]. However, increasing evidence indicates that aberrant EGFR signaling in NSCLC cells can also be caused by defects in the receptor internalization and degradation route [29]. We found that CMTM7 knockdown in NSCLC cells delays EGF-induced EGFR internalization and degradation. In accordance with these results, CMTM7 knockdown promotes NSCLC cell growth and migration. The regulation of EGFR internalization and degradation may thus be a mechanism for CMTM7 deletion-induced lung tumorigenesis. Currently, EGFR-targeted drugs have been approved for clinical use for patients with NSCLC. One potential mechanism of the action of EGFR-targeted drugs involves their ability to change EGFR internalization and degradation. For example, the EGFR-targeted antibody cetuximab can promote EGFR internalization and degradation, which has the potential to increase the survival of patients with advanced NSCLC [30]. Thus, a loss of CMTM7 may also affect sensitivity to EGFR-targeted therapies in NSCLC.

EGFR signaling not only occurs at the cell surface but also continues through the endosomal system. Moreover, sorting EGFR into different membrane-bound compartments plays a critical role in specifying a signaling outcome. For example, the Rab5 effector APPL1 specifically stimulates EGFR-AKT signaling from early endosomes [31]. MAPK scaffolding proteins, such as LAMTOR-2 and MP1/p14, regulate continued EGF-dependent MAPK signaling from late endosomes and lysosomes [32, 33]. Our finding that CMTM7 localizes with Rab5/EEA1-containing early endosomes but not with late endosome markers indicates a role of CMTM7 in early endosomes. Interestingly, CMTM7 knockdown selectively enhances AKT but not ERK pathway activation, which is stimulated by EGF. Because CMTM7 knockdown delays early endosome fusion, this effect might influence EGFR trafficking along the endocytic pathway. Thus, we speculate that CMTM7 may regulate the spatial and temporal distribution of EGFR in early endosomes, which is critical for EGF-stimulated PI3K/AKT activation.

Notably, the mean size of early endosomes is significantly reduced in CMTM7-knockdown cells, similar to the endosomes in cells in which endosomal fusion is inhibited by the overexpression of a dominant-negative Rab5 mutant (Rab5S34N) [34, 35]. In accordance with these results, CMTM7 knockdown reduces Rab5 activation. However, we did not identify the molecular mechanism underlying how Rab5 activation is controlled by CMTM7. Generally, Rab5 activation can be negatively regulated by GTPase-activating proteins (GAPs), which accelerate the intrinsic rate of GTP hydrolysis, and positively regulated by GEFs facilitating the GDP to GTP exchange [36]. The identified GEFs contain a highly conserved Vps9 domain that catalyzes nucleotide exchange on Rab5 [37]. Sequence and structure analysis of CMTM7 have not implicated it as a putative GEF. Rab5 activation is also regulated by other molecules in addition to GEFs and GAPs. Caveolin-1, a constitutive protein of the caveolae, controls Rab5 activation during caveolae-mediated endocytosis [38]. Plastin, an actin-bundling protein, controls endocytosis through the maintenance of Rab5 activation [39]. The class IA PI3-kinase p110β subunit activates Rab5 independently of its catalytic activation [40]. Based on these results, one possibility is that CMTM7 directly interacts with Rab5 in Rab5 activation. Another possibility is that CMTM7 affects the localization of different Rab5 GEFs and GAPs. We hope that further investigation will enable a better understanding of the role of CMTM7 in Rab5 activation.

Our results indicate that the loss of CMTM7 in NSCLC cells positively regulates EGFR signaling by decreasing Rab5 activation, thereby promoting tumor growth and migration. Multiple RTKs are often deregulated in NSCLC [41], especially HGF-hepatocyte growth factor receptor (cMet). Our data suggested that CMTM7 knockdown has no obvious effect on cMet stability and degradation (Supplementary Figure S3). As Rab5 controls the cellular fate of a wide spectrum of receptors, CMTM7 may be implicated in the internalization and degradation of other receptors, which needs further study. Taken together, our results indicate that CMTM7 is a new regulator of Rab5 activation in the intracellular trafficking network that controls EGFR-AKT signaling and may provide a potential target for the diagnosis and treatment of NSCLC.

## MATERIALS AND METHODS

### Cell lines, reagents and antibodies

Human NSCLC A549 and HCC827 cells were purchased from ATCC and grown in RPMI-1640 supplemented with 10% fetal bovine serum. The following reagents were used: human EGF (Peprotech, Rockville, NJ, USA), Texas Red-EGF, Lipofectamine 2000 (Invitrogen, Grand Island, NY, USA), the phosphatidylinositol-3-kinase inhibitor LY294002 (Cell Signaling Technology, Inc., Beverly, MA, USA), and polybrene (hexadimethrindibromide, Fluka, Buchs, Switzerland). The following antibodies were used: total-EGFR, phospho-EGFR^Tyr1173^, Rab5, Rabaptin-5 (Santa Cruz Biotechnology, Santa Cruz, CA, USA), His, Ubiquitin, total-AKT, phospho-AKT^Ser473^, total-ERK, phospho-ERK^Thr202/Tyr204^ (Cell Signaling Technology, Inc., Beverly, MA, USA), β-actin (Sigma-Aldrich, St Louis, MO, USA), and anti-CMTM7 mouse antibody was prepared and purified in our laboratory.

### Lentivirus-mediated shRNA knockdown of CMTM7

pGIPZ-lentiviral shRNAmir vectors targeting the human CMTM7 gene and nonsilencing pGIPZ control vector were purchased from Open Biosystems (Thermo Fisher Scientific, Inc.). The pGIPZ cloning vector contains Turbo GFP reporter and expresses a puromycin-resistant gene. Lentiviruses were produced by co-transducing the lentiviral vector plasmid containing shRNAmir and packaging plasmids psPAX2 and pLP/VSVG into 293T cells via Vigofect. At 72 h post-transfection, supernatants containing either the lentivirus expressing CMTM7 shRNA or control shRNA were collected, supplemented with 8 μg/ml of polybrene and filtered through a 0.45 μm filter unit. Target cells (A549 and HCC827 cells) were infected via 24 h incubation in virus-containing media at 37°C. The cells were kept under standard cell culture conditions with puromycin (1 μg/ml) for 3 weeks and then sorted by fluorescence-activated cell sorting. The highest green fluorescent protein-expressing cell population was selected for each cell line.

### Coimmunoprecipitation and immunoblotting

Coimmunoprecipitation assays were performed with control and CMTM7-knockdown A549 cells. Cells were cultured for 36 h and harvested in a buffer containing 20 mM Tris-HCl, pH 8.0, 150 mM NaCl, 2 mM EDTA, 10% glycerol, 0.5% Nonidet P-40, 1 mM dithiothreitol, 1 mM phenylmethylsulfonyl fluoride, 5 μg/ml leupeptin, 5 μg/ml aprotinin, and 5 μg/ml pepstatin. Whole-cell lysates (1000 μg) were incubated with indicated antibody for 12 h at 4°C and further incubated for another 2 h after the addition of pre-equilibrated protein-G Sepharose beads (GE, New York City, NY, USA). The sepharose beads were then washed three times with the same buffer at 4°C. Samples were then analyzed using western blotting according to the standard protocol described previously [6]. Signals were detected by LAS500 (GE, New York City, NY, USA).

### GST-R5BD pull-down assay

The GST-R5BD construct and pull-down assays were described previously [25]. Briefly, we cloned the cDNA of the Rab5-binding domain (R5BD, residues 739–862) of Rabaptin5 into the pGEX vector (GE, New York City, NY, USA). The resulting construct was termed pGEX/Rabaptin-5(R5BD), which expressed the fusion protein GST-R5BD in BL21-CodonPlus E. coli upon isopropyl β-d-thiogalactoside induction. GST-R5BD was then affinity purified with glutathione-Sepharose 4B resin (GE, New York City, NY, USA). For pull-down of cell lysates, cells in 10-cm plates were lysed in buffer containing 25 mM HEPES, pH 7.4, 100 mM NaCl, 1 mM CaCl_2_, 5 mM MgCl_2_, 1% NP-40, 10% glycerol, 1 mM DTT, 100 μM PMSF, and EDTA-free protease inhibitor cocktail. After centrifugation, supernatants were incubated with GST-R5BD beads at 4°C, washed with lysis buffer, boiled in 1× SDS sample buffer and subjected to immunoblotting.

### Cell growth assay

Cell growth assays were carried out as previously described [8].

### Soft agar colony-forming assay

A total of 0.6% agar (Sigma-Aldrich, St Louis, MO, USA) in RPMI-1640 medium supplemented with 10% fetal bovine serum was poured into 24-well culture plates (500 μl/well). After solidification, an equal amount of cell suspension (containing 200 cells) mixed with 0.3% soft agar was laid on top of the 0.6% agar in each well. After the agar solidified, 80 μl of 10% fetal bovine serum was added at regular intervals of 3–4 days. After 2–3 weeks, the colonies were fixed, stained with crystal violet and counted.

### Wound-healing assay

Wound-healing assays were conducted as previously described [42].

### Transwell migration assay

The migration assay was performed in a 24-well chemotaxis chamber (Neuro Probe, Cabin John, MD, USA) as previously described [7]. The top and bottom compartments of chambers were separated by polycarbonate filters (8 μm pore size). Briefly, serum-starved cells (2 × 10^5^ cells/ml) were resuspended in serum-free RPMI 1640 and added to the upper well of the chamber (5 × 10^4^ cells/well). Then, 10% fetal bovine serum was added to the lower well. The cells were incubated for 24 h at 37°C in a 5% CO_2_ humidified atmosphere. For EGF-induced migration, serum-starved cells were added to the upper well of the chamber (1 × 10^5^ cells/well). EGF (100 ng/ml) was placed in the bottom chamber and analyzed after 48 h. After incubation, non-migrated cells were scraped off and migrated cells were identified after fixing and staining. At least five random fields of vision per well were counted for the quantitation of migrated cells. The assay was repeated at least three times.

### *In vivo* metastasis assay

The animal study was approved by the institutional animal care and use committee of Peking University. CMTM7-knockdown or control A549 cells were trypsinized and suspended in PBS for tail vein injection. A total of 1.5 × 10^6^ cells in 200 μl of PBS were injected into the lateral tail vein of 8-week-old NOD-SCID mice (six mice per group). The mice were killed 6 weeks later, and excised lungs were fixed with Bouin-Hollande fixative for 24 h. Finally, the number of pulmonary metastatic nodules on the surface of lung was counted and the lungs were prepared for paraffin sections stained by hematoxylin and eosin.

### Analysis of surface EGFR using flow cytometry

For EGFR internalization, CMTM7-knockdown and control cells were serum starved for 16 h and then treated with 20 ng/ml EGF for the indicated times. The treated cells were trypsinized and centrifuged at 1600 rpm for 5 min at 4°C. The cells were washed with PBS, blocked and then incubated with anti-EGFR antibody, which recognizes the extracellular portion of the receptor, and an APC-conjugated goat anti-rabbit immunoglobulin. The cells (1 × 10^4^) were counted via flow cytometry (FACSCalibur) at an excitation of 488 nm and analyzed with CELLQuest software (BD Bioscience, USA).

### Confocal microscopy

Cells were washed with PBS, fixed and permeabilized in 3% paraformaldehyde containing 0.1% Triton X-100 for 30 min at 4°C. The cells were blocked and incubated with primary antibodies and then TRITC-conjugated secondary antibodies. The cells were washed twice with PBS and stained with DAPI for 10 min before being imaged with a TCS-SP laser-scanning confocal microscope with a 63× oil immersion lens (Leica Microsystems, Mannheim, Germany). Uptake of Texas Red-EGF was performed by first starving cells in serum-free medium for 16 h at 37°C. The cells were then incubated with cold serum-free medium containing 1000 ng/ml Texas Red-EGF for 30 min at 4°C. Endocytosis was initiated by replacing the ligand-binding medium with pre-warmed medium. At the end of each time point, the cells were rapidly chilled, washed, fixed, and then imaged as described above.

### Statistical analysis

The data were expressed as the mean ± s.d. Statistical analyses were performed using two-tailed Student's *t*-tests in Prism 5.0 (GraphPad Software, San Diego, CA, USA). Differences were considered significant when *P* < 0.05.

## SUPPLEMENTARY FIGURES


