# Supplementary material for: CMTM7 knockdown increases tumorigenicity of human non-small cell lung cancer cells and EGFR-AKT signaling by reducing Rab5 activation
Source: Oncotarget. 2015 Oct 26;6(38):41092–107. doi: 10.18632/oncotarget.5732 (PMC4747392; doi:10.18632/oncotarget.5732)
Supplement: Supplementary file 1 [file oncotarget-06-41092-s001.pdf]

SUPPLEMENTARY FIGURES

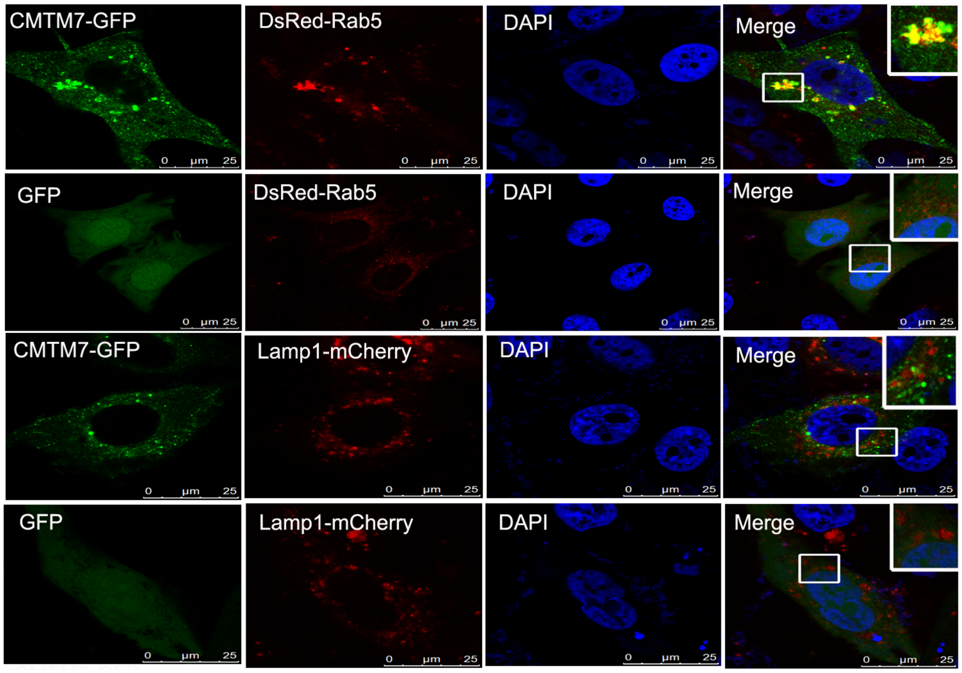

Supplementary Figure S1: CMTM7-GFP colocalizes with DsRed-Rab5, but not Lamp1-mCherry. A549 cells transiently transfected with CMTM7-GFP were co-transfected with DsRed-Rab5 or Lamp1-mCherry for 24 h. After fixation, cell images were captured via confocal microscopy. Nuclei are visualized by DAPI (blue). Bar, 25 μm.

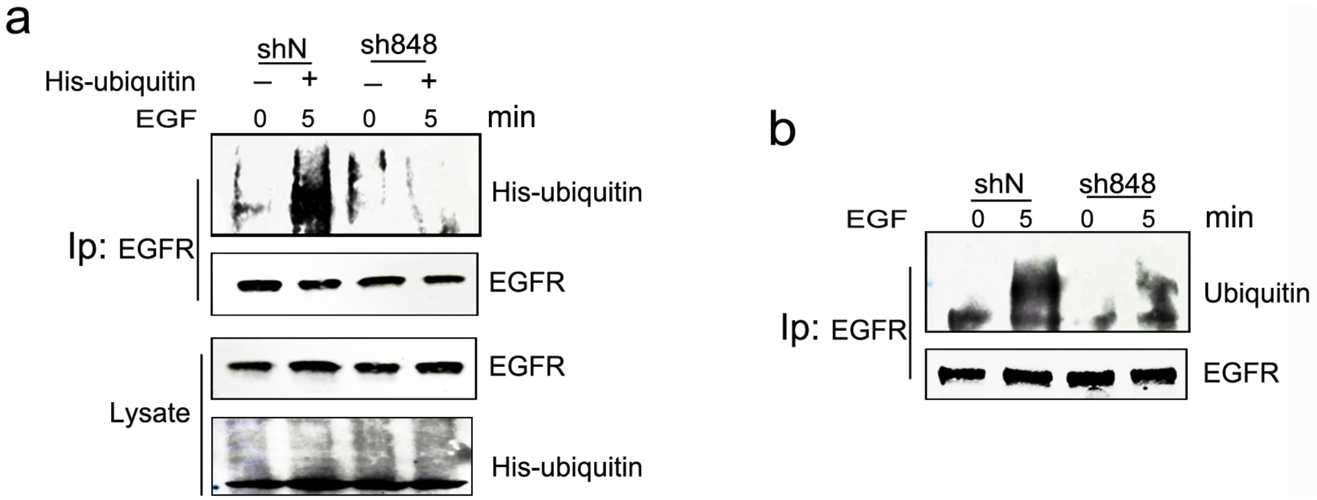

Supplementary Figure S2: CMTM7 knockdown decreases EGFR ubiquitination. a. Control and CMTM7-knockdown A549 cells were transiently transfected with His-ubiquitin. After 24 h post-transfection, cells were starved for 4 h and then stimulated with 100 ng/ml EGF for 5 min. Cell lysates were then subjected to immunoprecipitation with EGFR antibody and immunoblotting to detect ubiquitylated EGFR with His antibody. Equivalent expression of His-ubiquitin was confirmed by determining modification of proteins in total cell lysates by the tagged ubiquitin (bottom: a representative region of the blot is shown). **b.** Cells were starved for 4 h and then treated with100 ng/ml EGF for 5 min. Cell lysates were precipitated with anti-EGFR and analyzed for ubiquitylated EGFR levels with anti-ubiquitin antibody. Data are representative of three independent experiments.

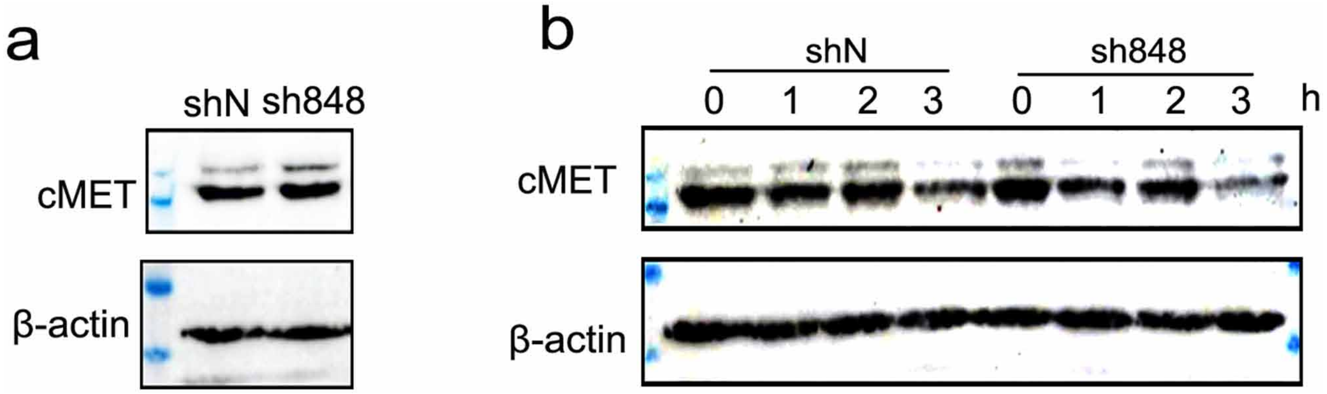

Supplementary Figure S3: CMTM7 knockdown has no obvious effect on cMet stability and degradation. a. Control and CMTM7-knockdown A549 cells were cultured in complete medium, and western blots of the cell lysates were probed with antibodies against the indicated proteins. Data are representative of three independent experiments. **b.** Control and CMTM7-knockdown A549 cells were pretreated with cycloheximide (CHX) (100 μg/ml) for 1 h prior to treatment with HGF (100 ng/ml) in the presence of CHX for the indicated times, and the cell lysates were subjected to immunoblotting with the indicated antibodies. Data are representative of three independent experiments.
